# Measuring success: The challenge of social protection in helping eliminate tuberculosis

**DOI:** 10.1371/journal.pmed.1002419

**Published:** 2017-11-07

**Authors:** Priya B. Shete, David W. Dowdy

**Affiliations:** 1 Division of Pulmonary and Critical Care Medicine, Zuckerberg San Francisco General Hospital, University of California, San Francisco, San Francisco, California, United States of America; 2 Global Tuberculosis Programme, World Health Organization, Geneva, Switzerland; 3 Department of Epidemiology and Biostatistics, Johns Hopkins University Bloomberg School of Public Health, Baltimore, Maryland, United States of America

## Abstract

In this Perspective on the research article by William Rudgard and colleagues, Priya Shete and coauthor discuss the challenges of measuring the impact of social protection programs such as cash transfers.

## Tuberculosis, poverty, and social protection

Tuberculosis (TB) disproportionately affects the poorest and most vulnerable individuals—people who face tremendous economic barriers to accessing TB diagnosis and treatment [1]. Even though TB clinical services are free to patients in most countries, the total financial burden of TB disease often amounts to over half of patients’ annual individual incomes [2]. These high costs reduce the probability of completing testing and initiating treatment [3], in turn increasing morbidity and mortality, fueling community TB transmission, and perpetuating poverty [4,5]. Interventions to alleviate social and economic risk (i.e., social protection interventions) therefore have great appeal in terms of their potential to alleviate not only poverty but also the burden of TB disease. Indeed, social protection interventions speak directly to a pillar of the World Health Organization’s (WHO) End TB Strategy: to ensure that no TB affected household incurs “catastrophic” costs in the course of their TB care [6]. And such interventions clearly improve the financial status of their beneficiaries [7]. But what (and how) would we measure to verify the claim that social protection actually improves TB care and control?

## Challenges in measuring the impact of social protection

If we are to demonstrate that social protection programs improve TB outcomes, we must first define an appropriate outcome measure that balances health status (e.g., morbidity, mortality, incidence) and economic vulnerability (e.g., income, education level, socioeconomic status). In the present issue of *PLOS Medicine*, William Rudgard and colleagues evaluate the association between cash transfer programs (a common form of social protection) and catastrophic costs, defined as total costs in excess of 20% of annual household income [8]. This outcome has emerged as a leading metric for analyzing the socioeconomic consequences of TB for several reasons. First, catastrophic costs (as opposed to catastrophic health expenditures [9]) incorporate both the direct costs associated with accessing TB care (for example, payments for transportation and childcare) and the indirect costs of TB disease, including income loss from time away from work. This is an important distinction given that income loss accounts for up to 60% of the total cost of TB for many patients [2]. Second, catastrophic costs have been associated with both adverse TB treatment outcomes (including death, treatment abandonment, treatment failure, or recurrence) and adverse economic practices such as dissaving (spending more than one earns in a given period) [10].

Rudgard and colleagues suggest that a TB-specific cash transfer approach, in which cash transfers are provided specifically to TB patients, has the potential to prevent patients with TB from suffering catastrophic costs more than TB-sensitive cash transfers, in which TB patients are eligible for cash transfers based on other measures of vulnerability but not specifically targeted by virtue of their TB status [8]. This important piece of evidence is only an early step towards demonstrating that a social protection intervention can improve TB outcomes. Additional research is necessary to determine whether the link between reduced catastrophic costs and improved TB outcomes results from factors associated with direct poverty alleviation and increased income or because reduced costs enable pro-health decision making about accessing TB care specifically.

Unfortunately, demonstrating that social protection improves a specific downstream health outcome is a very challenging task. Ecological studies have shown association between increases in national spending on social protection and decreases in TB burden [11]. However, establishing causal linkages between social protection interventions and TB outcomes requires the collection and analysis of targeted epidemiological and costing data aligned along a sound conceptual framework that describes the mechanisms between upstream social protection inputs and downstream TB outcomes in potentially context-specific ways [12,13]. If any criteria or steps along this pathway are not met—for example, if this pathway is not clearly delineated or is misspecified, catastrophic costs (or other measured quantity) may not map onto the underlying causal construct (for example, financial risk). Furthermore, if confounding is not adequately accounted for, implementing a social protection intervention may not improve clinical outcomes. Rudgard and colleagues demonstrate an association between TB-specific cash transfers and reduced catastrophic costs, but—as they rightly acknowledge—far more work is necessary before we can claim a causal link between any social protection scheme and improved TB outcomes, let alone the sustainability of effect and onward viability of such programs when implemented.

## A way forward

As we seek to build a stronger case for such social protection interventions improving TB outcomes, a number of elements must be addressed. First, we need better evidence to support consensus measured outcomes. In the case of catastrophic costs, for example, there are ongoing efforts to better measure TB-related costs across a variety of country contexts and to define the most appropriate threshold in terms of association with TB outcomes [14]. Not only will the sensitivity analyses done as part of these surveys help refine our definition of “catastrophic” costs, but they will provide the basis for improving our understanding of the types of social protection interventions best suited for improving TB patient outcomes. Second, we need stronger data, such as that derived from trials, ideally designed to support causal links between intermediate outcomes such as catastrophic costs and patient-centered outcomes such as TB morbidity and mortality. We have some data, for example, that link conditional cash transfers (CCTs) to improved economic outcomes [12] and data that link CCTs to improved TB outcomes [13]; however, we do not yet have clear data that demonstrate a clear mechanism by which improved economic outcomes lead to improved TB outcomes. Ideally, such data would come from a variety of epidemiological and economic contexts and have sufficient individual-level granularity to adjust for key confounders (e.g., malnutrition, drug resistance status, preexisting socioeconomic status, and severity of disease). Third, we need more data like that provided by the analysis of Rudgard and colleagues to illustrate the types of social protection interventions that are most likely to reduce financial risk (or other validated intermediate outcomes) among individuals with TB. And finally, we need empirical data on how to best operationalize social protection interventions either within the public health sector or more broadly as part of larger scale financial and social system reforms such as universal health coverage (UHC) [15].

Ultimately, if social protection is to help eliminate TB or any other disease of poverty, concerted effort is required between epidemiologists, clinicians, policy makers, economists, and health systems experts to create the evidence base necessary to demonstrate a causal link between the two. Progress in this regard is not only possible; it is necessary. The TB and global health communities must continue to integrate disease-specific health priorities within the larger health systems goals of UHC and global priorities outlined in the Sustainable Development Goals (SDG) agenda. Attempting to achieve these goals in the setting of limited data should reaffirm the importance of supporting research that is both methodologically rigorous and politically relevant. Only through such a coordinated approach can we hope to eliminate specific diseases such as TB while also achieving sustainable multisector development targeting those populations most affected by diseases of poverty and the poverty of disease.

## Abbreviations

CCT: conditional cash transfer
SDG: Sustainable Development Goals
TB: tuberculosis
UHC: universal health coverage
WHO: World Health Organization
